# Transcriptome Analysis of Porcine Thymus following Porcine Cytomegalovirus Infection

**DOI:** 10.1371/journal.pone.0113921

**Published:** 2014-11-25

**Authors:** Xiao Liu, Zhiwen Xu, Ling Zhu, Shan Liao, Wanzhu Guo

**Affiliations:** 1 Animal Biotechnology Center, College of Veterinary Medicine, Sichuan Agricultural University, Ya' an, China; 2 Key Laboratory of Animal Disease and Human Health, College of Veterinary Medicine, Sichuan Agricultural University, Ya' an, China; University of Regensburg, Germany

## Abstract

Porcine cytomegalovirus (PCMV) is a major immunosuppressive virus that mainly affects the immune function of T lymphocytes and macrophages. Despite being widely distributed around the world, no significantly different PCMV serotypes have been found. Moreover, the molecular immunosuppressive mechanisms of PCMV, along with the host antiviral mechanisms, are still not well characterized. To understand the potential impact of PCMV on the function of immune organs, we examined the transcriptome of PCMV-infected thymuses by microarray analysis. We identified 5,582 genes that were differentially expressed as a result of PCMV infection. Of these, 2,161 were upregulated and 3,421 were downregulated compared with the uninfected group. We confirmed the expression of 13 differentially expressed immune-related genes using quantitative real-time RT-PCR, and further confirmed the expression of six of those cytokines by western blot. Gene ontology, gene interaction networks, and KEGG pathway analysis of our results indicated that PCMV regulates multiple functional pathways, including the immune system, cellular and metabolic processes, networks of cytokine-cytokine receptor interactions, the TGF-β signaling pathway, the lymphocyte receptor signaling pathway, and the TNF-α signaling pathway. Our study is the first comprehensive attempt to explore the host transcriptional response to PCMV infection in the porcine immune system. It provides new insights into the immunosuppressive molecular mechanisms and pathogenesis of PCMV. This previously unrecognized endogenous antiviral mechanism has implications for the development of host-directed strategies for the prevention and treatment of immunosuppressive viral diseases.

## Introduction

Porcine cytomegalovirus (PCMV) is a member of the genus *Roseolovirus*, subfamily *Betaherpesvirinae*, and family *Herpesvirus*. It has a 128,367 bp linear double-stranded DNA genome, containing 79 open reading frames (ORFs), and its viral particle diameter is 150–200 nm [1]. Viruses of genus *Cytomegalovirus* are widely distributed in nature and have strict host specificity; for example, PCMV only infects pigs. PCMV has been documented worldwide, with pig farms in Japan, Europe, North America, and China having an average infection rate of 90% [1]–[2]. Interestingly, no distinct PCMV serotypes have been identified. PCMV spreads by both vertical and horizontal transmission, and a recent study showed that PCMV was present in pig semen, indicating that the virus can spread through mating [3].

PCMV can remain latent in adult pigs, but active infection causes fatal systemic failure in piglets less than 3 weeks of age. The clinical symptoms of infected piglets include pneumonia and inclusion body rhinitis, and there is a high mortality rate. PCMV-infected sows are prone to abortion, with pathological changes including edema in the heart, lungs, lymph nodes, and mesocolon [2].

In recent years, because of the shortage of human organ donors, xenotransplantation has become an emergency alternative option. Because pigs are the major donors for xenotransplantation, a variety of porcine viruses have become a threat to the human recipients. Porcine endogenous retroviruses and porcine lymphotropic herpesvirus 1 and 2 have previously been identified as major concerns for organ transplantation; however, the ubiquitous nature of herpesviruses, including PCMV, means that these viruses are now a major focus in the development of xenotransplantation technology [1], [4], [5].

PCMV inhibits host immune function and defense mechanisms, particularly the action of T lymphocytes. Like porcine reproductive and respiratory syndrome virus, PCMV uses alveolar macrophages as target cells, and a recent study showed that PCMV infection can promote the occurrence of porcine reproductive and respiratory disease [6].

Microarray technology is used to monitor target molecules by detecting the intensity of hybridization signals, and it is capable of both high-throughput and high sensitivity. It can detect transcriptional level changes in entire host genomes in response to pathogens, allowing a more detailed understanding of the molecular mechanisms of host-pathogen interactions during viral infection [10]–[12]. Although a series of transcriptome profiles have been generated for the host in response to herpesvirus family infections, a specific transcriptome analysis of the host following PCMV infection that focuses on the immunosuppressive molecular mechanisms of PCMV is still lacking [7]–[9].

The current research used the Agilent Pig 4×44K Gene Expression Microarray v2 to comprehensively analyze differences in the transcriptomes of the thymuses of pigs infected with PCMV compared with those of control pigs. The expression of a group of immune-related genes identified by the microarrays was confirmed by quantitative RT-PCR (qPCR) and western blot. The results of this study further both our understanding of the genes involved in the porcine immune response to PCMV and the pathogenesis of PCMV, and they will contribute to the prevention and treatment of immunosuppressive viral diseases.

## Results

### Confirmation of PCMV infection

**Figure 1:**
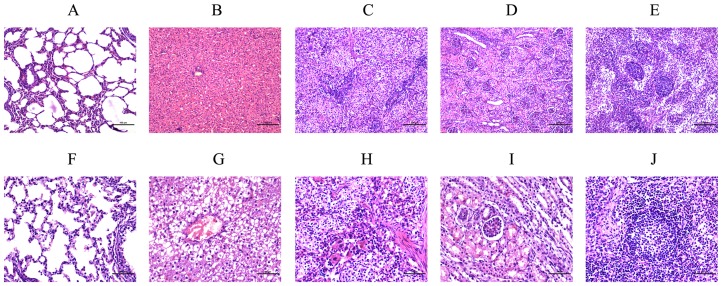
Pathological section examination. A–E: Control porcine (A) lung, (B) liver, (C) spleen, (D) kidney, and (E) thymus tissue sections. F–J: PCMV-infected porcine (F) lung, (G) liver, (H) spleen, (I) kidney, and (J) thymus tissue sections.

All piglets inoculated with the PCMV SC strain showed clinical symptoms of weakness, fever, rhinitis, and conjunctivitis from 7 days post inoculation (dpi). The PCMV infection induced pulmonary edema, pleural effusion, splenic infarction, and surface bleeding of the kidneys (Fig. S1). Biopsies of PCMV-infected piglets showed dilatation and congestion of alveolar capillaries and significantly swollen hepatocytes, while the hepatic sinusoids were completely absent. We also found increased numbers of splenic macrophages, decreased numbers of lymphocytes, and granular degeneration of tubular epithelial cells in PCMV-infected piglets (Fig. 1). In contrast, we observed no significant pathological changes in the uninfected piglets (Fig. 1). Serological tests showed that all PCMV-inoculated piglets were antibody-positive, while the uninfected piglets showed a negative serum antibody response. We also quantified the PCMV RNA in the tissues using qPCR. The viral loads of the thymuses from the two dead PCMV-infected piglets were 4.72×10^7^ copies/mg and 8.43×10^7^ copies/mg. No viral load was detected in the thymuses from the control group. These results confirmed successful infection of the PCMV-inoculated pigs.

### Transcriptome analysis of the immune organs in response to PCMV infection

**Figure 2:**
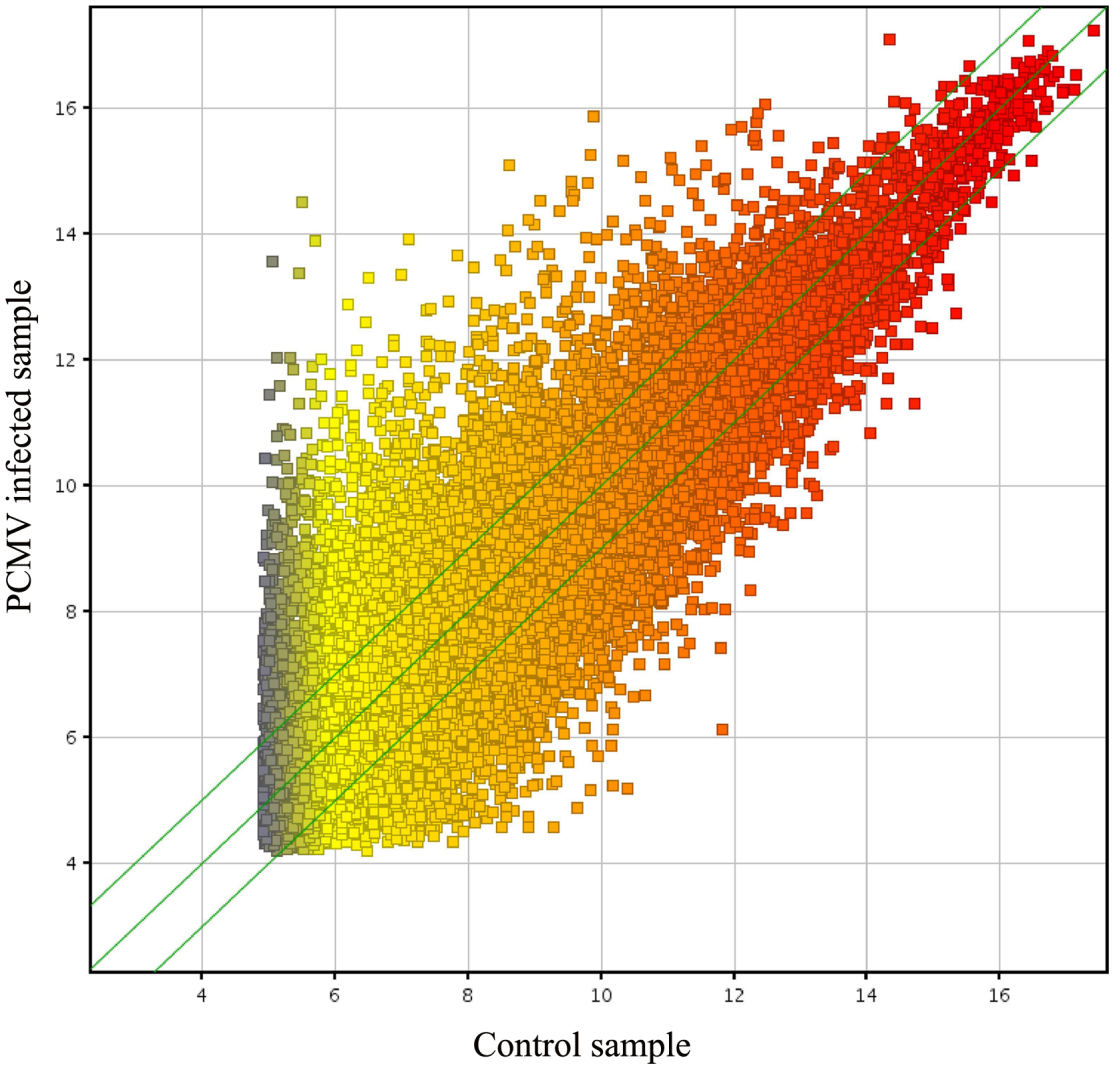
Scatter plot of the microarray data. The data from the microarray are graphed on a scatter plot to visualize variations (or reproducibility) in gene expression between arrays. The values on the X and Y axes of the scatter plot are the normalized signal values for the samples (log2 scaled). The green lines are fold-change lines (the default fold-change value is 2.0). The expression of the genes above the top green line or below the bottom green line differed more than two-fold between the infected and control samples.

**Figure 3:**
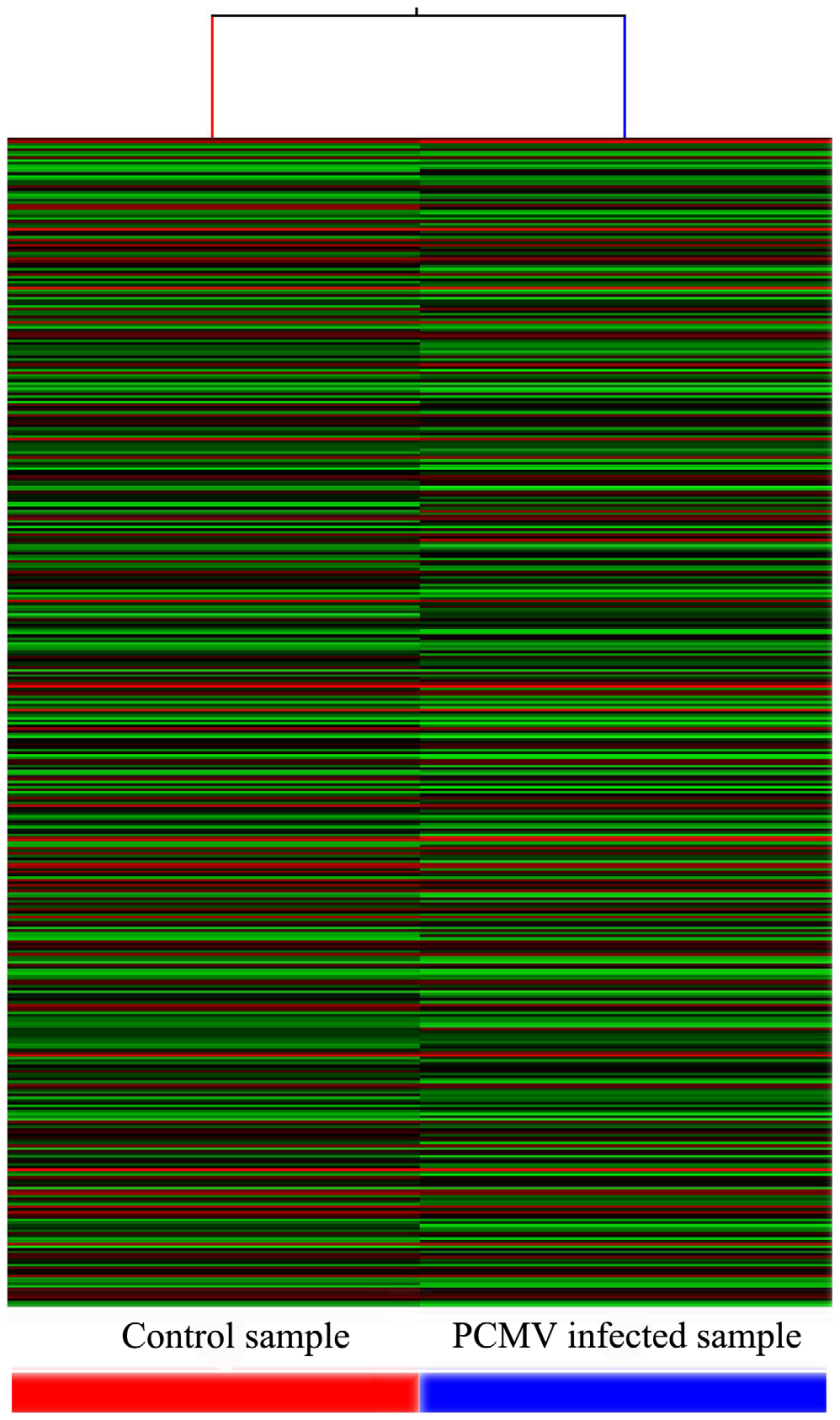
Heat map and hierarchical clustering of the microarray data. Hierarchical clustering was used to analyze the gene expression data by arranging the samples into groups based on their expression levels. The dendrogram shows the relationships among the gene expression patterns in the samples. The red line indicates high relative expression and the green line indicates low relative expression.

The microarray analysis showed a significant host response to PCMV infection compared with the uninfected controls. To confirm differential expression of the host genes identified by the microarray, we performed fold-change filtering between the two samples (default threshold was fold change ≧2.0). We identified 15,527 genes in the microarray analysis (Table S1) (NCBI GEO Accession: GSE59115, ID: 200059115). PCMV infection resulted in 2,161 upregulated and 3,421 downregulated genes compared with the uninfected group (Table S2). The results from the quality assessment of the gene data after filtering are shown in Figs. 2–3 and Fig. S2.

**Figure 4:**
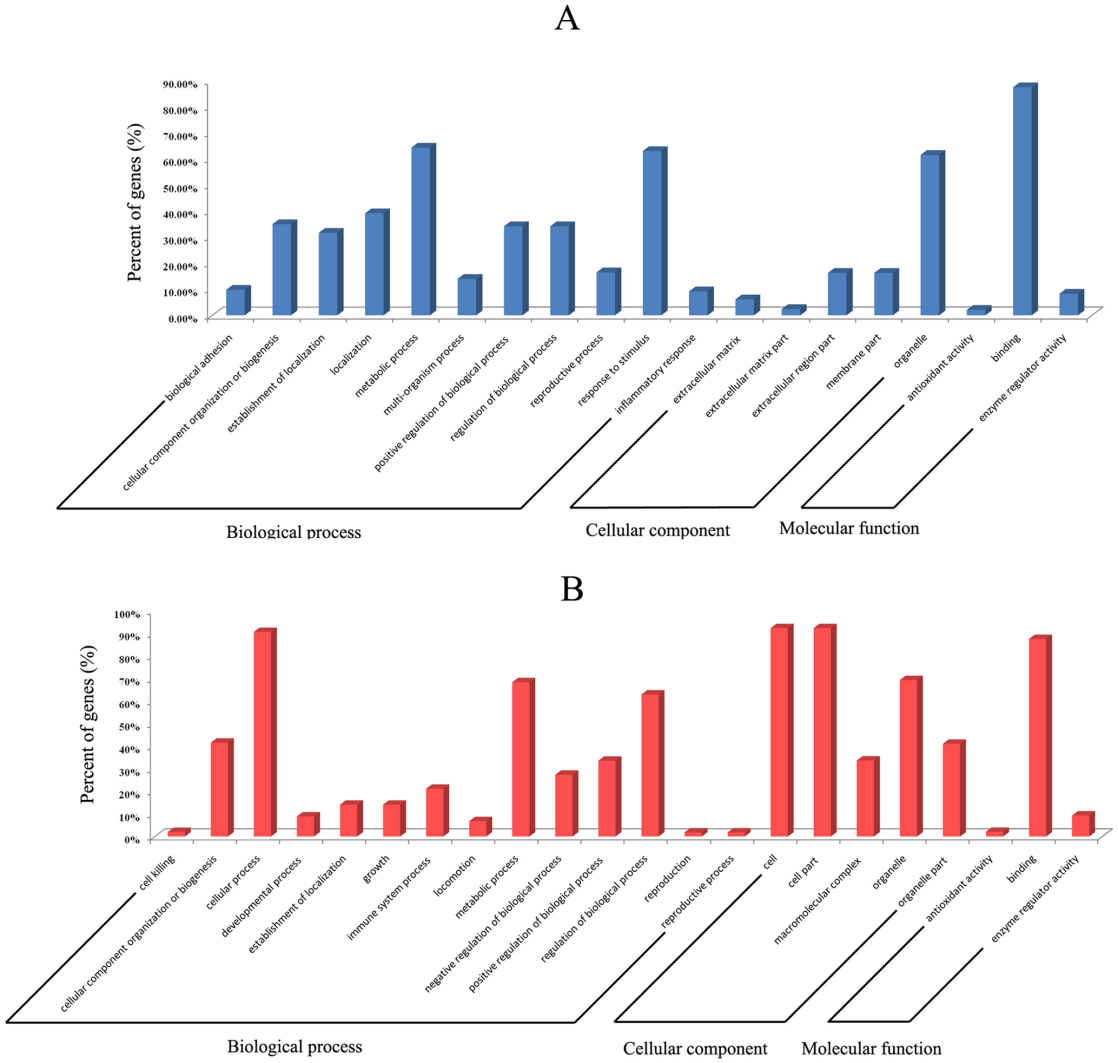
GO annotation of the differentially expressed genes. A GO analysis to categorize the differentially expressed genes into GO categories was performed. The P values indicate the significance of the GO term enrichment in the list of differentially expressed genes (P≤0.05 is considered significant). (A) GO annotation of the upregulated genes. (B) GO annotation of the downregulated genes. For other enriched GO terms, please see Table S3.

The differentially expressed genes were functionally annotated based on gene ontology (GO) (www.geneontology.org). The results showed that the differentially expressed genes were associated with cellular components, molecular functions, and biological processes. The GO category analysis revealed that the upregulated genes fell into the following categories: inflammatory responses (e.g. interleukin 1 beta), stimulus responses (e.g. bone morphogenetic protein 2 and transforming growth factor beta 2/3), metabolic processes (e.g. thrombospondin receptor and serine dehydratase), and protein binding (e.g. cytoplasmic cochaperone 1). The main GO categories for the downregulated genes were cellular processes (including cellular metabolic processes, cellular processes involved in reproduction, regulation of cellular processes, and organelle organization, e.g. aprataxin), immune system processes (e.g. Sp3 transcription factor, interleukin 6, and toll-like receptors), metabolic processes (e.g. aprataxin, mutL homolog 1, and cathepsin D), and regulation of biological process (e.g. upstream transcription factor 1 and toll-like receptor 2) (Fig. 4 and Table S3).

To further elucidate the function of the differentially expressed genes in the porcine thymuses after PCMV infection, a Kyoto Encyclopedia of Genes and Genomes (KEGG) analysis was performed. In total, 143 over-represented pathways were identified, suggesting that these signaling pathways are regulated by the differentially expressed genes during virus infection (Table S4). The differentially expressed genes were mainly associated with the chemokine signaling pathway, the cytokine-cytokine receptor interaction pathway, the cytokine signaling pathway, the T-cell receptor signaling pathway, and the apoptosis signaling pathway.

### STRING analysis

**Figure 5:**
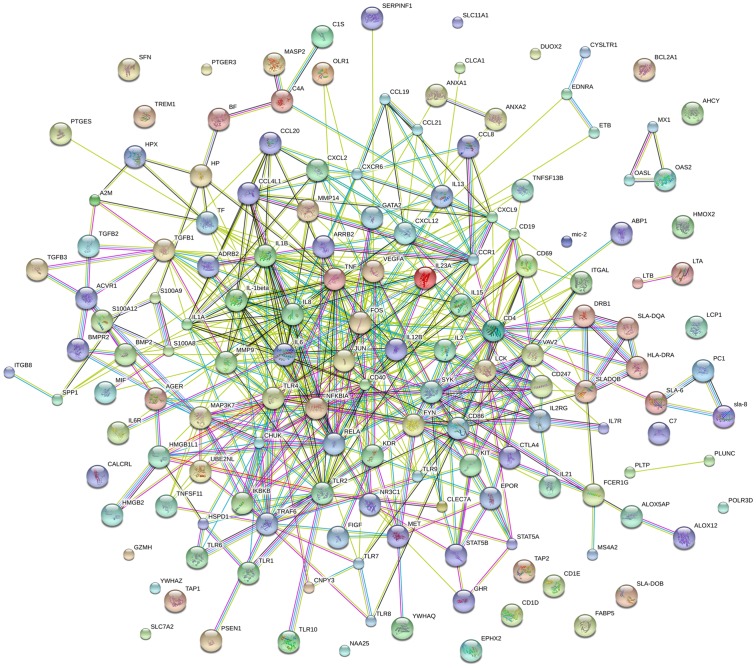
Interaction networks of the differentially expressed genes. The STRING database was used to predict the relationships between the differentially expressed immune-related genes. The lines of different colors represent the types of evidence upon which the associations are based: green, neighborhood evidence; red, gene fusion evidence; blue, co-occurrence evidence; black, coexpression evidence; purple, database evidence; cyan, text-mining evidence; yellow, homology evidence.

The STRING (Search Tool for the Retrieval of Interacting Gene/Proteins) online software was used to predict the direct (physical) and indirect (functional) interactions between the differentially expressed immune-related proteins that were mainly related to the inflammatory response, the immune response and its regulation, and cytokine signaling pathways. The functional protein association networks (Fig. 5) indicated that most of the genes involved in these processes were interrelated. For example, the genes encoding tumor necrosis factor (TNF), tumor necrosis factor ligand superfamily member-13b, interleukin-15 (IL-15), IL-6, IL-8, IL-1α, IL-1β, IL-6 receptor, transforming growth factor beta 1 (TGF-β), chemokine (C-X-C motif) ligand 9 (CXCL9), chemokine (C-X-C motif) ligand 12 (CXCL12), and chemokine (C-C motif) ligand 20 (CCL20) were associated with multiple signaling pathways. The genes encoding chemokine (C-C motif) ligand 4-like 1 (CCL4L1), CD4, CD86, CD40, toll-like receptor 4 (TLR4), TLR9, and kinase insert domain receptor were related with one another, and the genes encoding IL-8, IL-6, IL-1β, CD4, and IL-1α were also interrelated. However, some of the genes were not linked to the association network, including those encoding triggering receptor expressed on myeloid cells 1, MHC class I related antigen 2, and lymphocyte cytosolic protein 1.

### Confirmation of microarray data by qPCR and western blot

**Figure 6:**
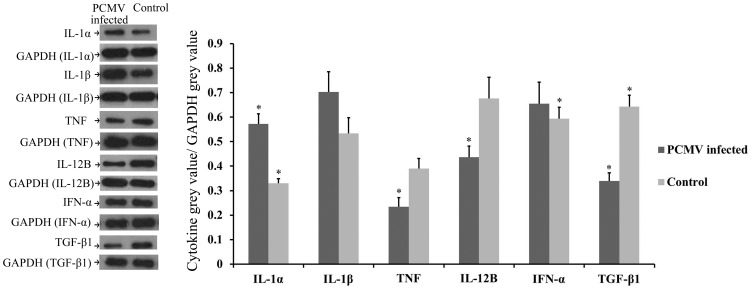
Western blot of six of the differentially expressed genes. Western blot of IL-1α, IL-1β, IFN-α, IL-12B, TNF and TGF-β1 in samples from control and PCMV-infected pig thymuses. (* denotes P<0.05).

To evaluate the role of specific host immune genes in PCMV infection, we selected 13 immune-related genes for further analysis by qPCR (IL-1α, IL-1β, IL-2, IL-7, IL-8, IL-12, IL-15, IL-7R, IFN-α, TNF, TNF-SF10, TGF-β1, and TGF-β-R1). We additionally confirmed 6 of those cytokines by western blot (IL-1α, IL-1β, IL-12B, IFN-α, TNF, and TGF-β1). We found that the detected level of gene expression was relatively consistent between qPCR, western blot, and microarray analysis for all the genes tested (Table 1, Table S5, and Fig. 6).

**Table 1:** Real-time qRT–PCR of 13 differentially expressed genes.

Genes	GeneBank Accession	qRT-PCR fold-change (Infected/control)	Microarray fold-change (Infected/control)
IL1a	NM_214029	+2.22	+2.09
IL1b	NM_214055	+1.13	+3.91
IL2	NM_213861	−1.05	−2.98
IL7	NM_214135	+1.13	+1.47
IL8	X61151	+3.13	+4.15
IL12	NM_214013	−1.64	−4.19
IL15	NM_214390	+1.88	+2.36
IL-7R	NM_001146128	−1.25	−3.41
TNF	NM_214022	−1.08	−2.01
TNFSF10	NM_001024696	+1.38	+1.11
TGF-β1	NM_214015	−1.40	−3.11
TGF-β-R1	NM_001038639	+1.60	+1.08
IFN-α	NM_001166310	+1.31	+1.31

qRT-PCR results of 13 immune-related genes in thymuses from PCMV-infected pigs compared with those of normal pigs. “+” and “–” indicate upregulated and downregulated genes, respectively. qRT-PCR Ct threshold: 0.015. Fold-change cut-off: 2.0.

## Discussion

The interaction between a virus and its host is determined by the host's immune response. Like human cytomegalovirus (HCMV), PCMV suppresses the immune system, especially the cell-mediated immune response [13]; however, there has been no research into the immune-evasion mechanism of PCMV until now. The thymus is one of the most important central immune organs, which is mainly composed of lymphocytes, macrophages, dendritic cells, epithelial cells, and reticular cells. The thymus is the main site of the proliferation, differentiation, and maturation of immune cells. It plays a crucial regulatory role in the development and immune function of peripheral immune organs. Changes in the thymus transcriptome during infection with an immunosuppressive virus directly reflect the impact of the virus on the immune function of host, which is the reason we chose the thymus as the site for our microarray experiments [14].

This is the first comprehensive analysis of the transcriptomes of porcine immune organs during PCMV infection. Using microarray technology, we detected 5,582 differentially expressed genes. Thirteen of the immune-related genes were confirmed with RT–qPCR and six of those cytokines were further confirmed by western blot. The differential expression of a range of immune-related genes in the porcine thymuses suggests that the infection pressure of PCMV affects the immune process of the host.

The results of GO annotation and KEGG analyses showed that several of the differentially expressed genes are involved in cellular signaling pathways, including the genes for T-cell receptor (TCR), TLR, NF-κB, B-cell receptor, TNF, p53, and TGF-β, and others are involved in the cytokine–cytokine receptor interaction signaling pathways.

TCR signaling in response to antigen recognition plays a crucial role in the adaptive immune response [15]. In this study, the expression levels of most genes involved in the TCR signaling pathway were downregulated, including those encoding cytotoxic T-lymphocyte-associated protein 4, CD4/8, lymphocyte-specific protein tyrosine kinase, CD3ε, CD3δ, E3 ubiquitin protein ligase, CD40 ligand, NF-κB, IL-2, TNF-α, inducible T-cell co-stimulator, growth factor receptor-bound protein 2, and pyruvate dehydrogenase kinase isozyme 1. This suggests that the TCR signaling pathway is significantly inhibited during infection by PCMV.

TGF-β is an immunosuppressive cytokine, and it plays an important regulatory role in several cellular processes and immune functions. The TGF-β signaling pathway is also involved in apoptosis, cell differentiation, and growth [16]. Recent research has shown that the proliferation of activated T cells is suppressed by apoptosis and the release of TGF-β1 during HCMV infection [17]. Human immunodeficiency virus and hepatitis C virus infections also activate the TGF-β signaling pathway [18]. In the present study, the results of a GO annotation analysis showed that several of the upregulated genes are associated with the TGF-β signaling pathway, including genes encoding TGF-β2, TGF-β3, inhibitors of DNA binding 1/2/4, activin RI, decorin, and inhibin βB. This suggests that the activated TGF-β signaling pathway may function in PCMV infection as it does during infections by other immunosuppressive viruses.

The interferons (IFNs) are proteins with antiviral and immunoregulatory activities, and they have been shown to play essential roles in the host responses to viruses of the family *Herpesviridae* [19]-[22]. An HCMV-derived IL-10 homolog was shown to suppress the TLR-induced expression of IFN-α/β genes in infected plasmacytoid dendritic cells, thus inhibiting the antiviral and immunoregulatory activities of the host [23]–[24]. However, in the present study, the expression of the genes encoding IFN-α/β were not significantly upregulated in porcine thymuses after PCMV infection.

The TLR family is the first line of defense against infectious agents, and it plays a fundamental role in the innate immune response. These proteins identify and monitor pathogen-associated molecular patterns and also mediate the expression of the cytokines that are necessary for immunity. Currently, roles for TLRs in infections by members of the family *Herpesviridae* have been reported. For example, activated TLR7/8 signaling contributes to the inhibition of bovine alpha herpesvirus replication [25]–[26]. Apart from TLR3 and TLR4, the expression levels of the genes encoding most of the TLRs, including TLR1, TLR2, TLR6, TLR7, TLR8, and TLR10, were significantly downregulated after PCMV infection. This implies that PCMV inhibits the innate immune response by interrupting the expression of TLRs.

Chemokines also play an important role in the inflammatory response. Recent research has shown that infection by viruses of the family *Herpesviridae* promotes the expression of inflammatory cytokines and chemokines [27]–[28]. In this study, the expression levels of the genes encoding IL-8, CXCL2, and chemokine receptor CCR1 were upregulated during PCMV infection, suggesting that IL-8 exerts an effect similar to its effect during infection by other *Herpesviridae* family viruses.

The chemokine receptor CCR7 and its ligands (CCL20/CCL19) play important regulatory roles in adaptive immune functions, including regulatory T-cell and memory T-cell functions and thymocyte development [29]. In this study, the expression levels of *CCL4*, *CCL20*, *CCL19*, *CCL8*, *CCL21*, *CXCL9*, and *CXCL12* were reduced after PCMV infection, suggesting that PCMV affects the adaptive immune functions of the host by regulating the secretion of chemokines.

Apoptosis is an important defense mechanism of organisms, and viruses can evade identification by their hosts' immune systems by directly or indirectly inhibiting the apoptosis process. Virus-encoded proteins can also induce host cell apoptosis during the release of the viral particles in the late stage of infection [30]–[32]. The caspase protein family is essential for the modulation of apoptosis. Caspase 3 and caspase 10 are encoded by the *CASP3* and *CASP10* genes, respectively, and play central roles in the execution-phase of host cell apoptosis [33]–[35]. In this study, the expression levels of *CASP3* and *CASP10* were significantly reduced compared with those in the control group.

The apoptosis regulator-2 (BCL-2) family of proteins is also critical for apoptosis, with its members having either anti-apoptotic or pro-apoptotic functions [36]. Recent research showed that the viral G-protein-coupled receptor (vGPCR) inhibits apoptosis of endothelial cells by upregulating BCL-2 expression during infection by Kaposi sarcoma-associated herpesvirus [37]. The expression levels of BCL-2-related protein A1 (encoded by *BCL2A1*), BCL-2-associated gene 6 (*BAG6*), and BCL-2-associated transcription factor 1 (*BCLAF1*) were significantly downregulated after PCMV infection.

The cytokines of the TNF superfamily play important roles in the regulation of cell development, physiological processes, and the host innate and adaptive immune responses to viral pathogens, including in the inhibition of viral replication, the regulation of immune genes, the development of peripheral lymphoid organs, and the differentiation of lymphocytes. The apoptotic and nonapoptotic signaling pathways can be activated after TNF receptors engage with their cognate ligands, thereby regulating the host's antiviral activity [38]. Viruses can also regulate the host's immune processes by interrupting the TNF signaling pathway [39]. In this study, the expression levels of the majority of genes involved in the TNF signaling pathway, including those encoding TNF, IL-6, CCL20, caspase 10, caspase 3, NF-κB, and TGF-β-activated kinase 1 (TAK1), were significantly downregulated after viral infection. This implies that the TNF signaling pathways and apoptotic processes are inhibited by PCMV infection, which is conducive to the replication of the virus.

Like the TNF/TNFR signaling pathway, the FASL/FAS pathway is also involved in apoptosis-associated signal transduction and caspase activation. However, there were no significant changes in the expression of genes involved in the FASL/FAS pathway after PCMV infection. In summary, we have shown that PCMV affects apoptosis by regulating the expression of a range of apoptosis-related genes, and in this way, facilitates latent viral infection [40].

The central immune organs are the major sites for the proliferation and differentiation of immune cells, whereas the peripheral lymphoid organs are the main sites of the immune response. Identification of the interaction between immune-related proteins in the immune organs and viral pathogens is critical for understanding the viral immunosuppressive mechanism. Our results confirm that PCMV not only directly or indirectly affects the function of the immune cells and organs, but also inhibits the host immune function by regulating the expression of multiple cytokines. This comprehensive analysis of the transcriptome of porcine thymus during PCMV infection extends our understanding of the adaptive immune response to PCMV infection and of the immune-evasion mechanisms of PCMV. This knowledge will contribute to the prevention and treatment of immunosuppressive viruses.

## Materials and Methods

### Ethical statement

The animal welfare standards adhered to in this study were established according to internationally agreed and science-based principles of the World Organization for Animal Health (OIE). All experiments were carried out in accordance with China Animal Welfare Legislation and were approved by the Sichuan Agricultural University Committee for Ethics in the Care and Use of Laboratory Animals.

### Animals, viral infection, and detection

The PCMV Sichuan (SC) strain and three-week-old male Yorkshire piglets were provided by the Animal Biotechnology Center of Sichuan Agricultural University (Ya'an, China). The 10 piglets were divided into two groups of five pigs each and maintained under controlled temperature and humidity. Serum samples collected from all 10 piglets were confirmed as PCMV antibody-negative before the start of the experiment, as described previously [41]. Each pig in the first group was inoculated with 5 ml of 10^9^ PFU/ml PCMV SC strain by intramuscular (3 ml) and intranasal (2 ml) injection at the same time, and each pig in the control group was injected with 5 ml of RPMI-1640 nutrient solution (Thermo Fisher Scientific, Waltham, UK) in the same way. The anti-PCMV antibody levels in the serum samples collected from the infected and control pigs at 13 dpi were determined as described previously [41]. Two piglets died during the viral challenge experiment (13 and 14 days dpi), and the thymus from each dead pig was collected immediately. The thymuses from two control pigs were also collected at the same time points to serve as controls.

Quantitative RT–PCR (RT–qPCR) was used to detect the PCMV viral load in the thymuses of two dead PCMV-infected piglets, and samples from the two control piglets were also tested by RT-qPCR as controls, as described previously [42]. To observe the pathological changes in the infected porcine tissues, paraffin sections were examined as described previously [43]. The sera, lymph nodes, spleens, and thymuses of the remaining infected and control pigs were collected at 14 dpi, and all the tissues were immediately frozen in liquid nitrogen and stored at –80°C until further analysis.

### RNA extraction

All thymuses from infected and control piglets were combined into a single infected group and a single control group, respectively, before RNA extraction. Total RNA was extracted from the thymuses of the PCMV-infected piglets and control piglets using TRIzol Reagent (Invitrogen, Shanghai, China), according to the manufacturer's instructions. The integrity of the RNA was assessed with electrophoresis on a denaturing agarose gel. A NanoDrop ND-1000 spectrophotometer (NanoDrop Inc., Wilmington, DE, USA) was used to accurately measure the RNA concentrations.

### Microarray analysis

The Pig 4×44K Gene Expression Array was manufactured by Agilent. The whole pig genome oligo microarray is a broad-range array that represents well-known pig genes and transcripts. When coupled to Agilent's probe selection and robust validation processes, this array delivers improved data quality and less redundant gene coverage.

### RNA labeling and array hybridization

Samples were labeled and array hybridization was performed according to the Agilent One-Color Microarray-Based Gene Expression Analysis protocol (Agilent Technology). The total RNA from each sample was linearly amplified and labeled with Cy3–UTP. The labeled cRNAs were purified with the RNeasy Mini Kit (Qiagen) according to the manufacturer's instructions. The concentration and specific activity of the labeled cRNAs (pmol Cy3/µg cRNA) were measured with a NanoDrop ND-1000 spectrophotometer. Each labeled cRNA (1 µg) was fragmented by the addition of 11 µl of 10× Blocking Agent and 2.2 µl of 25× Fragmentation Buffer and was then heated at 60°C for 30 min. Next, 55 µl of 2× GE Hybridization Buffer was added to dilute the labeled cRNA. The hybridization solution (100 µl) was dispensed onto the gasket slide, which was assembled onto the gene expression microarray slide. The slides were incubated for 17 h at 65°C in an Agilent Hybridization Oven. The hybridized arrays were washed, fixed, and scanned with the Agilent DNA Microarray Scanner.

### Data analysis

The Agilent Feature Extraction software (version 11.0.1.1) was used to analyze the acquired array images. Quantile normalization and subsequent data processing were performed with the GeneSpring GX v11.5.1 software package (Agilent Technologies). After the quantile normalization of the raw data, genes that were flagged as “Detected” in at least two of two samples (“All Targets Value”) were selected for further analysis. Differentially expressed genes were identified with fold-change filtering. Hierarchical clustering was performed with the Agilent GeneSpring GX software (version 11.5.1).

GO analysis, which is a functional analysis that associates differentially expressed genes with GO categories, was performed on our microarray data. The GO categories are derived from Gene Ontology (www.geneontology.org), which contains three structured networks of defined terms that describe the attributes of gene products.

A pathway analysis of the differentially expressed genes was performed using the standard enrichment computation method and based on the latest KEGG database. This analysis allowed us to determine the biological pathways that are significantly enriched in the differentially expressed genes. The P value (EASE-score, Fisher-Pvalue, or Hypergeometric-Pvalue) denotes the significance of the pathway correlated to the conditions. The interaction networks of the differentially expressed genes were predicted using the STRING 9.1 database (http://www.string-db.org/).

### RT–qPCR

RT–qPCR was used to confirm the transcriptional levels of specific immune-related genes with the SYBR Green PCR Core Reagents Kit (Applied Biosystems, Foster City, CA, USA) on the ABI Prism 7900 Sequence Detection System (Applied Biosystems), according to the manufacturer's instructions. The RT–qPCR primers used to detect IL-1α, IL-1β, IL-2, IL-7, IL-8, IL-12, IL-15, IL-7R, TNF, TNF-SF10, TGF-β1, TGF-β-R1, and IFN-α are shown in Table 2. The amplification conditions were: 94°C for 10 min, followed by 40 cycles of 95°C for 10 s and 60°C for 60 s. The conditions used to establish the melting curves of the PCR products after the amplification reaction were: 95°C for 10 s, 60°C for 60 s, and 95°C for 15 s, followed by heating from 60°C to 90°C.

**Table 2:** Primers used for RT–qPCR.

Genes	GeneBank Accession	Forward primers (5′-3′)	Reverse primers (5′-3′)	Product length
GAPDH	NM_001206359	ATGGTGAAGGTCGGAGTGAAC	CTCGCTCCTGGAAGATGGT	235
IL1a	NM_214029	TGAAGTGTTGACAGGCCGTAT	TGGTCCTCCCAAGATTGTTATG	250
IL1b	NM_214055	AGGAAGTGATGGCTAACTACGGT	GCTGGATGCTCCCATTTCTC	126
IL2	NM_213861	GAGCCATTGCTGCTGGATTT	GTAGCCTGCTTGGGCATGTAA	110
IL7	NM_214135	AGTGACTATGGGCGGTGAGA	GCTACTGGCAACAGAACAAGG	142
IL8	X61151	CTATGCCTCATTCCTGTGCT	AGAACAACGTGCATGGGAC	121
IL12	NM_214013	CCTGGGAAAGTCCTGTCGTG	GCAGATTTTGGGAGTGGTTGA	282
IL15	NM_214390	GCGATGAAGTGCTTTCTCCTG	TACTCAATGGACGATAAACTGCTG	122
IL-7R	NM_001146128	CTCCTTCTCGTGCTACAGTCA	ACTCAGGCAATTTATGTCCAA	139
TNF	NM_214022	CACGCTCTTCTGCCTACTGC	TCCCTCGGCTTTGACATTG	163
TNFSF10	NM_001024696	TCCAGCAGAGCCAATAGGTTA	GTGTTGTTGAGCCTTTGGGT	66
TGF-β1	NM_214015	ACTACTACGCCAAGGAGGTCA	TCTGCCCGAGAGAGCAATAC	157
TGF-β-R1	NM_001038639	CATAACCGTACAGTCATTCACCA	CGACCTTTGCCAATACTTTCT	200
IFN-α	NM_001166310	GACTCCATCCTGGCTGTGA	ATGACTTCTGCCCTGACGA	104

### Western blot analysis

All thymuses from infected and control piglets were combined into a single infected group and a single control group, respectively, and were lysed in Enhanced RIPA Lysis Buffer (Leagene, Beijing, China) containing 1 mM phenylmethanesulfonyl fluoride. After being resolved by sodium dodecyl sulfate-polyacrylamide gel electrophoresis (SDS-PAGE), the proteins were transferred to a nitrocellulose membrane (Bio-Rad, Shanghai, China). The following antibodies were used for protein detection: primary antibodies - rabbit polyclonal anti-IL-1α, anti-IL-1β, anti-IL-12B, anti-TNF, anti-IFNα, and anti-TGF-β1, and secondary antibody horseradish peroxidase conjugated anti-rabbit (abcam, Shanghai, China). Blots were visualized by the ChemiScope 3100 chemiluminescence imaging system (CLINX, Shanghai, China) according to the manufacturer's guidelines.

## Conclusion

This is the first comprehensive analysis of the transcriptomes of a porcine immune organ (thymus) during PCMV infection. Using microarray technology, 5,582 differentially expressed genes were detected, which were associated with the inflammatory response, the immune response, immunoregulation, and cytokine-mediated signaling pathways. We confirmed 13 differentially expressed immune-related genes using quantitative real-time RT-PCR and further confirmed 6 of those cytokines by western blot. This study provides new insight into the immunosuppressive molecular mechanisms and pathogenesis of PCMV. The results presented here will contribute to the prevention and treatment of immunosuppressive viral diseases.

## Supporting Information

Figure S1 **Pathological changes in PCMV-infected porcine organs.** The lungs of (A) PCMV-infected and (B) control pigs.
(TIF)

Figure S2 **Box plot of the intensity distributions from infected and control samples.** A box plot comparing the distributions of the intensities of the infected and control samples after normalization.
(TIF)

Table S1 **Gene expression profiles.**
(XLS)

Table S2 **Genes differentially expressed by control and PCMV-infected pig thymuses.** Genes were considered to be significantly upregulated or downregulated if the fold-change was ≧2.0 or ≤-2.0. Fold change is the ratio of normalized intensities between the infected and control samples. +: upregulated, -: downregulated.
(XLS)

Table S3 **GO functional enrichment annotations for the differentially expressed genes.**
(XLSX)

Table S4 **KEGG pathway annotations for the differentially expressed genes.**
(XLSX)

Table S5 **Real-time RT–PCR of 13 differentially expressed genes.**
(XLSX)

## References

[1] Gu W, Zeng N, Zhou L, Ge X, Guo X, et al (2014) Genomic organization and molecular characterization of porcine cytomegalovirus,. Virology. 460:165–172. 25010282 10.1016/j.virol.2014.05.014

[2] Edington N, Wrathall A, Done J (1988) Porcine cytomegalovirus (PCMV) in early gestation. Vet microbiol 17:117–128. 2845635 10.1016/0378-1135(88)90003-x

[3] Liu X, Liao S, Zhu L, Xu Z, Zhou Y (2013) Molecular Epidemiology of Porcine Cytomegalovirus (PCMV) in Sichuan Province, China: 2010–2012. PloS one 8:e64648. 23762243 10.1371/journal.pone.0064648 PMC3675093

[4] Scobie L (2010) Porcine pathogens and xenotransplantation: PERV and beyond!. Xenotransplantation 17:118–118.

[5] Mueller NJ, Livingston C, Knosalla C, Barth RN, Yamamoto S, et al (2004) Activation of porcine cytomegalovirus, but not porcine lymphotropic herpesvirus, in pig-to-baboon xenotransplantation. J Infect Dis 189:1628–1633. 15116299 10.1086/383351

[6] Yoon K-J, S Henry, Zimmerman J (1996) Isolation of porcine cytomegalovirus from a swine herd with PRRS. Vet. Med. 91(8):779–784.

[7] Van Beurden SJ, Gatherer D, Kerr K, Galbraith J, Herzyk P, et al (2012) Anguillid herpesvirus 1 transcriptome. J Virol 86:10150–10161. 22787220 10.1128/JVI.01271-12 PMC3446616

[8] Lisnic VJ, Cac MB, Lisnic B, Trsan T, Mefferd A, et al (2013) Dual analysis of the murine cytomegalovirus and host cell transcriptomes Reveal new aspects of the virus-host cell Interface. PLoS pathog 9:e1003611. 24086132 10.1371/journal.ppat.1003611 PMC3784481

[9] Ebrahimi B, Dutia BM, Roberts KL, Garcia-Ramirez JJ, et al (2003) Transcriptome profile of murine gammaherpesvirus-68 lytic infection. J Gen Virol 84:99–109. 12533705 10.1099/vir.0.18639-0

[10] Levine AJ, Horvath S, Miller EN, Singer EJ, Shapshak P, et al (2013) Transcriptome analysis of HIV-infected peripheral blood monocytes: Gene transcripts and networks associated with neurocognitive functioning. Journal of neuroimmunology 265:96–105. 24094461 10.1016/j.jneuroim.2013.09.016 PMC3855455

[11] Zhao H, Dahlo M, Isaksson A, Syvanen A-C (2012) The transcriptome of the adenovirus infected cell. Virology 424:115–128. 22236370 10.1016/j.virol.2011.12.006

[12] Veloso A, Warr GW, Browdy CL, Chapman RW (2011) The transcriptomic response to viral infection of two strains of shrimp (Litopenaeus vannamei). Dev Comp Immunol 35:241–246. 20955731 10.1016/j.dci.2010.10.001 PMC7103212

[13] Grey G, Nelson J (2008) Identification and function of human cytomegalovirus microRNAs. J Clin Virol 41:186–191. 18243786 10.1016/j.jcv.2007.11.024 PMC2706132

[14] Miller F (2002) The discovery of thymus function and of thymus-derived lymphocytes,. Immunol Rev. 185:7–14. 12190917 10.1034/j.1600-065x.2002.18502.x

[15] Sloan D, Jerome R (2007) Herpes simplex virus remodels T-cell receptor signaling, resulting in p38-dependent selective synthesis of interleukin-10. J Virol 81:12504–12514. 17804501 10.1128/JVI.01111-07 PMC2169026

[16] Chai Y, Ito Y, Han J (2003) TGF-β signaling and its functional significance in regulating the fate of cranial neural crest cells. Crit Rev Oral Biol M 14:78–88. 12764071 10.1177/154411130301400202

[17] Wang Y, Liu Y, Zhang Y, Peng L, Ma J, et al (2006) The role of the CD95, CD38 and TGF-β 1 during active human cytomegalovirus infection in liver transplantation. Cytokine 35:193–199. 17027281 10.1016/j.cyto.2006.08.001

[18] Kim MS, Kim S, Myung H (2014) Degradation of AIMP1/p43 Induced by Hepatitis C Virus E2 Leads to Upregulation of TGF-β Signaling and Increase in Surface Expression of gp96. PloS one 9:e96302. 24816397 10.1371/journal.pone.0096302 PMC4015952

[19] Orange JS, Biron CA (1996) Characterization of early IL-12, IFN-alphabeta, and TNF effects on antiviral state and NK cell responses during murine cytomegalovirus infection. J Immunol 156:4746–4756. 8648121

[20] Presti RM, Pollock JL, Dal Canto AJ, O'Guin AK, Virgin HW (1998) Interferon γ regulates acute and latent murine cytomegalovirus infection and chronic disease of the great vessels. J Exp Med 188:577–588. 9687534 10.1084/jem.188.3.577 PMC2212470

[21] Heise MT, Pollock JL, O'Guin A, Barkon ML, Bromley S, et al (1998) Murine Cytomegalovirus Infection Inhibits IFNγ-Induced MHC Class II Expression on Macrophages: The Role of Type I Interferon. Virology 241:331–344. 9499808 10.1006/viro.1997.8969

[22] Biron CA (1998) Role of early cytokines, including alpha and beta interferons (IFN-α/β), in innate and adaptive immune responses to viral infections. Semin Immunol pp. 383–390 Elsevier. 9799713 10.1006/smim.1998.0138

[23] Chang W, Barry PA, Szubin R, Wang D, Baumgarth N (2009) Human cytomegalovirus suppresses type I interferon secretion by plasmacytoid dendritic cells through its interleukin 10 homolog. Virology 390:330–337. 19524994 10.1016/j.virol.2009.05.013 PMC2747589

[24] Lee GC, Yi HA, Lee CH (2006) Stimulation of interferon-β gene expression by human cytomegalovirus via nuclear factor kappa B and phosphatidylinositol 3-kinase pathway. Virus Res 117:209–214. 16545883 10.1016/j.virusres.2005.08.018

[25] Van Gent M, Braem SG, de Jong A, Delagic N, Peeters JG, et al (2014) Epstein-Barr Virus Large Tegument Protein BPLF1 Contributes to Innate Immune Evasion through Interference with Toll-Like Receptor Signaling. PLoS pathog 10:e1003960. 24586164 10.1371/journal.ppat.1003960 PMC3930590

[26] Lima GK, Zolini GP, Mansur DS, Freire Lima BH, Wischhoff U, et al (2010) Toll-like receptor (TLR) 2 and TLR9 expressed in trigeminal ganglia are critical to viral control during herpes simplex virus 1 infection. Am J Pathol 177:2433–2445. 20864677 10.2353/ajpath.2010.100121 PMC2966801

[27] Ho J, Liang R, Srivastava G (1999) Differential cytokine expression in EBV positive peripheral T cell lymphomas. Mol Pathol 52: 269. 10.1136/mp.52.5.269 PMC395709 10748876

[28] Desloges N, Schubert C, Wolff MH, Rahaus M (2008) Varicella-zoster virus infection induces the secretion of interleukin-8. Med Microbiol Immun 197: 277–284. 10.1007/s00430-007-0060-3 17909856

[29] Comerford I, Harata-Lee Y, Bunting MD, Gregor C, Kara EE, et al (2013) A myriad of functions and complex regulation of the CCR7/CCL19/CCL21 chemokine axis in the adaptive immune system. Cytokine Growth F R 24:269–283. 10.1016/j.cytogfr.2013.03.001 23587803

[30] Fink SL, Cookson BT (2005) Apoptosis, pyroptosis, and necrosis: mechanistic description of dead and dying eukaryotic cells. Infect Immun 73:1907–1916. 15784530 10.1128/IAI.73.4.1907-1916.2005 PMC1087413

[31] Marshall WL, Yim C, Gustafson E, Graf T, Sage DR, et al (1999) Epstein-Barr virus encodes a novel homolog of the bcl-2 oncogene that inhibits apoptosis and associates with Bax and Bak. J Virol 73:5181–5185. 10233985 10.1128/jvi.73.6.5181-5185.1999 PMC112567

[32] Westendorp MO, Frank R, Ochsenbauer C, Stricker K, Dhein J, et al (1995) Sensitization of T cells to CD95-mediated apoptosis by HIV-1 Tat and gp120. Nature 375:497–500. 7539892 10.1038/375497a0

[33] Wang J, Chun HJ, Wong W, Spencer DM, Lenardo MJ (2001) Caspase-10 is an initiator caspase in death receptor signaling. Proceedings of the National Academy of Sciences 98:13884–13888. 10.1073/pnas.241358198 PMC61136 11717445

[34] Dorstyn L, Read SH, Quinn LM, Richardson H, Kumar S (1999) DECAY, a novel Drosophila caspase related to mammalian caspase-3 and caspase-7. J Biol Chem 274:30778–30783. 10521468 10.1074/jbc.274.43.30778

[35] Miller DK (1997) The role of the caspase family of cysteine proteases in apoptosis. Semin Immunol pp. 35–49 Elsevier 10.1006/smim.1996.0058 9106306

[36] Vogler M, Butterworth M, Majid A, Walewska RJ, Sun X-M, et al (2009) Concurrent up-regulation of BCL-XL and BCL2A1 induces approximately 1000-fold resistance to ABT-737 in chronic lymphocytic leukemia. Blood 113:4403–4413. 19008458 10.1182/blood-2008-08-173310

[37] Montaner S, Sodhi A, Pece S, Mesri EA, Gutkind JS (2001) The Kaposi's sarcoma-associated herpesvirus G protein-coupled receptor promotes endothelial cell survival through the activation of Akt/protein kinase B. Cancer Res 61:2641–2648. 11289142

[38] Benedict CA (2003) Viruses and the TNF-related cytokines, an evolving battle. Cytokine Growth F R 14:349–357. 10.1016/s1359-6101(03)00030-3 12787571

[39] Aravalli RN, Hu S, Rowen TN, Gekker G, Lokensgard JR (2006) Differential apoptotic signaling in primary glial cells infected with herpes simplex virus 1. J Neurovirol 12:501–510. 17162665 10.1080/13550280601064921

[40] Contini P, Ghio M, Merlo A, Poggi A, Indiveri F, et al (2005) Apoptosis of antigen-specific T lymphocytes upon the engagement of CD8 by soluble HLA class I molecules is Fas ligand/Fas mediated: evidence for the involvement of p56^lck^, calcium calmodulin kinase II, and calcium-independent protein kinase C signaling pathways and for NF-KB and NF-AT nuclear translocation. J Immunol 175:7244–7254. 16301629 10.4049/jimmunol.175.11.7244

[41] Liu X, Zhu L, Shi X, Xu Z, Mei M, et al (2012) Indirect-blocking ELISA for detecting antibodies against glycoprotein B (gB) of porcine cytomegalovirus (PCMV). J Virol Methods 186:30–35. 22981981 10.1016/j.jviromet.2012.08.024

[42] Fryer JF, Griffiths PD, Fishman JA, Emery VC, Clark DA (2001) Quantitation of porcine cytomegalovirus in pig tissues by PCR. J Clin Microbiol 39:1155–1156. 11230447 10.1128/JCM.39.3.1155-1156.2001 PMC87893

[43] O'Malley JT, Merchant SN, Burgess BJ, Jones DD, Adams JC (2008) Effects of fixative and embedding medium on morphology and immunostaining of the cochlea. Audiol Neuro-Otol 14:78–87. 10.1159/000158536 PMC2631091 18827478

