# Supplementary figures and images for: Transcriptome Analysis of Porcine Thymus following Porcine Cytomegalovirus Infection
Source: PLoS One. 2014 Nov 25;9(11):e113921. doi: 10.1371/journal.pone.0113921 (PMC4244220; doi:10.1371/journal.pone.0113921)

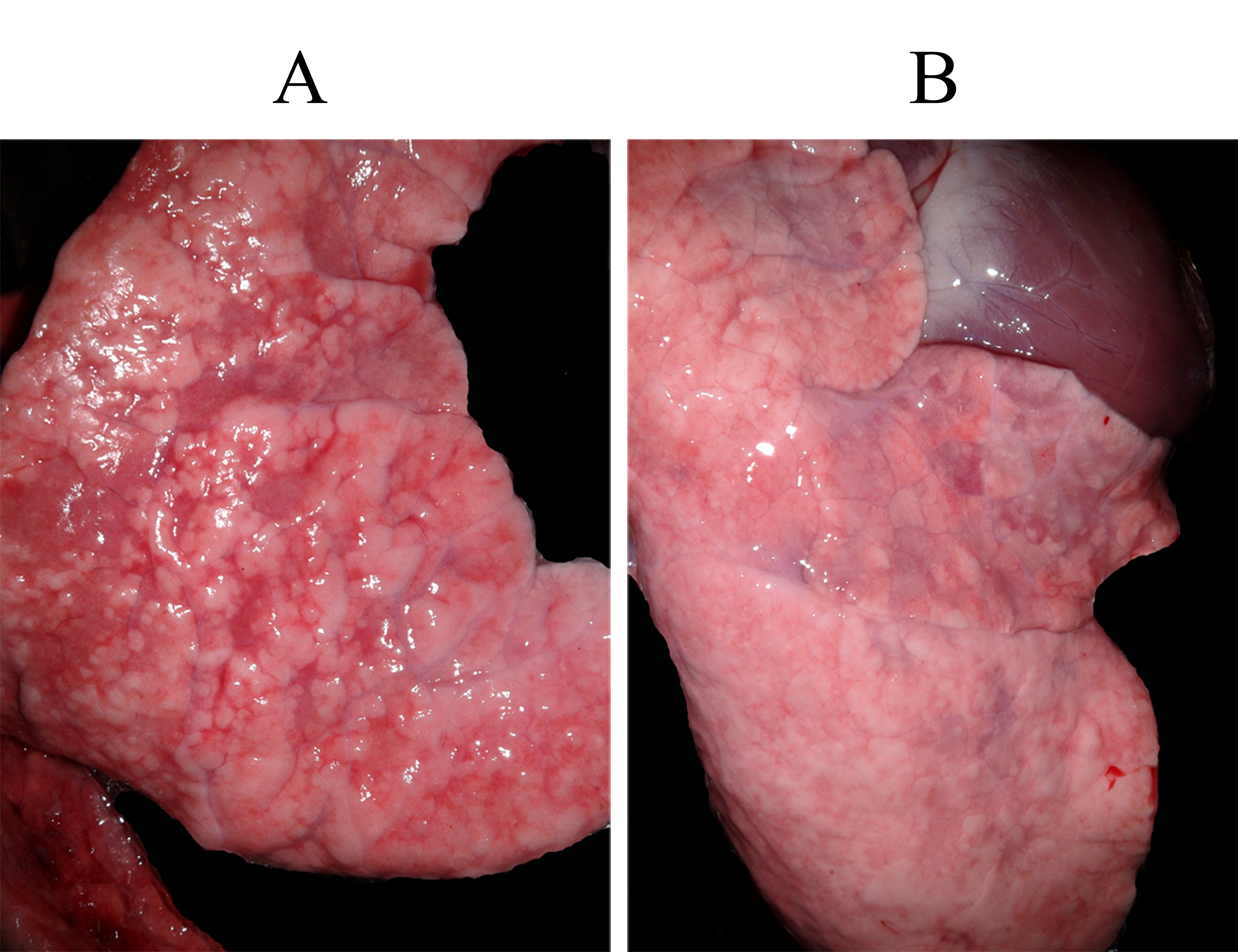

Supplement: Figure S1 — Pathological changes in PCMV-infected porcine organs. The lungs of (A) PCMV-infected and (B) control pigs. (TIF) [file pone.0113921.s001.tif]

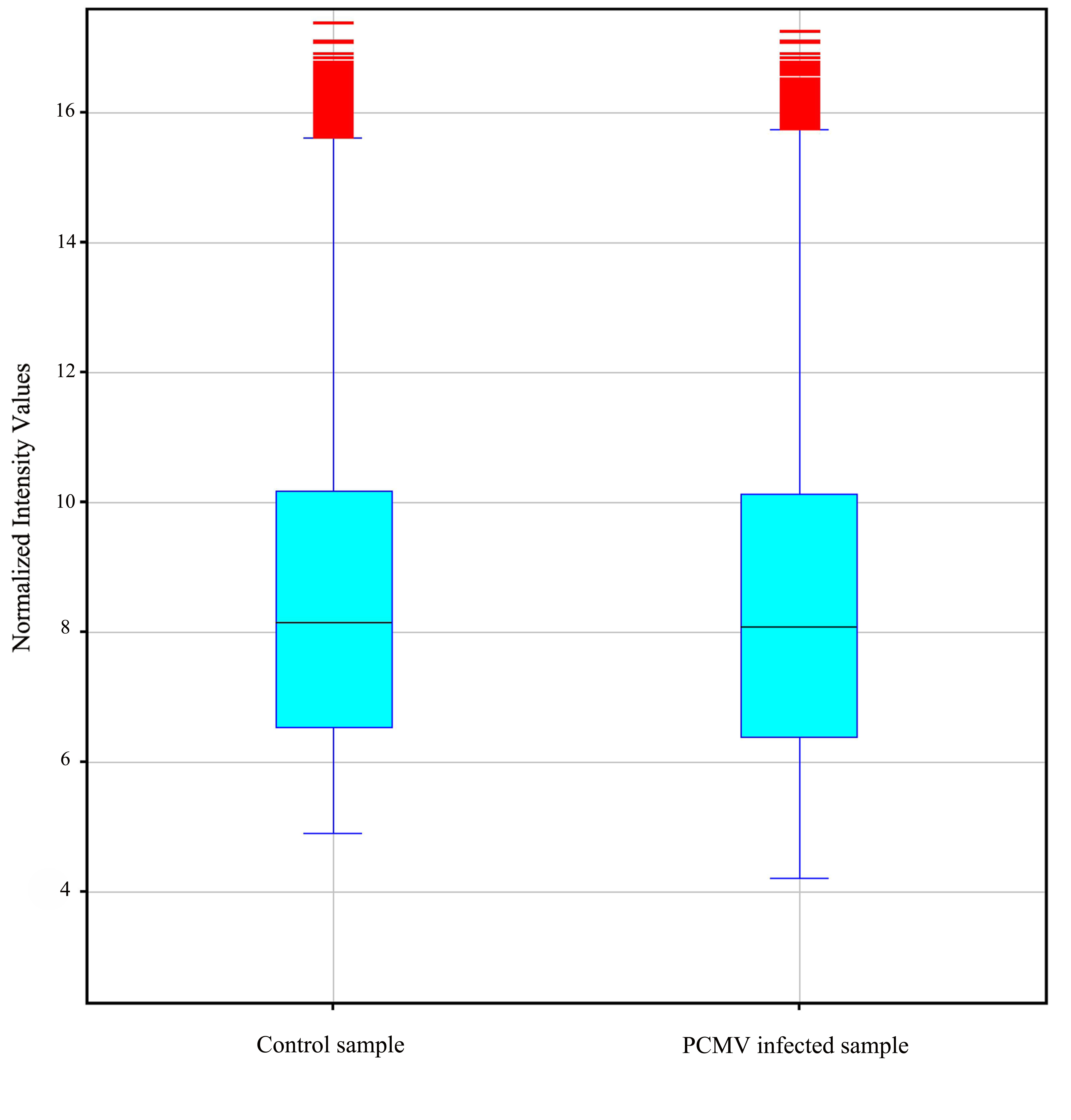

Supplement: Figure S2 — Box plot of the intensity distributions from infected and control samples. A box plot comparing the distributions of the intensities of the infected and control samples after normalization. (TIF) [file pone.0113921.s002.tif]
